# The Obesity Pandemic—Whose Responsibility? No Blame, No Shame, Not More of the Same

**DOI:** 10.3389/fnut.2020.00002

**Published:** 2020-01-31

**Authors:** Elliot M. Berry

**Affiliations:** Braun School of Public Health, Hebrew University-Hadassah Medical School, Jerusalem, Israel

**Keywords:** obesity, prevention and self-management, parental and individual responsibility, political correctness, sociotype, nine-level action plans, public health and medicine

“*Imprisoned in every fat man, a thin man is wildly signaling to be let out”*

Cyril Connolly, rotund British writer, (1903–1974)

Can we say this, let alone think it, in 2020?

## Introduction

No one doubts the economic costs of obesity, estimated at 5–14% of health expenditure for 2020–2050 (1), but there is disagreement whether fatness is considered a disease (2) or a behavioral risk factor, similar to smoking, alcohol and substance abuse that may lead to a disease (3). Current opinion also emphasizes social determinants and equity, thereby moving away from personal responsibility concepts (4). Although recent competencies for medical training do recommend chronic disease models and personalized obesity management care plans (5), there is no mention of topics such as *self-management, locus of control* or *responsibility*. It is not clear whether this is because they are considered unimportant or because they are not politically correct—yet, they are critical components for chronic disease management such as diabetes, post-transplant care and obesity (6). The paradigm chosen has major implications for prevention and treatment strategies.

That the obesity pandemic continues unabated represents a catastrophic failure of government policy, public health, and medicine—but not *only* of these domains. Table 1 lists different levels of responsibility incorporating the sociotype ecological framework (6). Levels 1–4 represent context and systems; levels 5–7 society and interpersonal relationships; and levels 8–9 parental and intrapersonal health and psychological make-up. Personal editorial input from a leading obesity journal suggested that “at this point in time, … emphasis on the personal responsibility (rather than the biology), would only add to the stigmatization of obesity in general.” I disagree—and concerning the biology—see the beginning of the discussion section. Without considering aspects of responsibility, obesity management is severely compromised. There are at least two sides to personal responsibility: medicalizing obesity, which reduces it, and parental supervision, which emphasizes it, since fat children are at high risk for adult obesity (7). Finally, I suggest an integrated nine-level approach that eschews political correctness and hopefully is not more of the same.

**Table 1:** The nine multilevel responsibilities and examples of strategies required for tackling obesity.

	**Level of responsibility**	**Some action strategies**
1	National	Prioritize the problem with focus on health inequalities and the socially disadvantaged; Health/Environment impact assessments; Legislation—“sin” taxes, ban sugary drinks in schools and junk food advertising
2	Food system	Equitable, sustainable food security; Food industry to reformulate products with healthier ingredients; Marketing transparency; Food labeling
3	Education system	Nutrition, cooking, activity, and lifestyle education throughout school years; Infrastructure for regular exercise; Monitoring weight and eating disorders?; School lunches, Eating manners
4	Medical system	Health care system; Motivational, non-judgmental, shared-decision approach to obesity counseling; Insurance coverage; Personal example
5	Public health	Health literacy for breast feeding, nutrition, and lifestyle choices according to social context; Positive deviance examples
6	Local authority, municipality	Promoting safe, leptogenic environments for all ages, especially in deprived residential areas; Farmers markets; Healthy Cities initiatives; Milan urban food policy pact
7	Society, community	Family and communal meals; Cultural attitudes (norms, values, social support); Peer groups; Binge eating; Social media and influencers; Role models; Gender sensitivity; Civil society; Media
8	Parental	Intra-uterine effects; Breast feeding; Parenting skills; Nutrition and lifestyle education; Regulation of screen time; Personal example
9	Individual	Lifestyle choices; Self-management, self-efficacy skills; Stress management; Coping strategies; Learned behaviors; Personality; Sense of humor
		Biology: Genetics; Epigenetics; Metabolic efficiency; Endocrine status; Energy homeostasis; Brain reward neurocircuitry

## Why Medicalize Obesity Management?

Insurance regulations may be part of the reason to promote a medical rather than a personal choice paradigm. This has two consequences. First, medicalizing the diagnosis helps ensure continuing insurance coverage for the severely obese. Second, it guarantees long-term reimbursement for the treating physicians. Unfortunately, such medicalization externalizes locus of control (8), decreases incentives to change lifestyle behaviors and deters self-management necessary to take active responsibility for weight regulation, noting that intelligence has little to do with self-control.

Personal preferences determine what, and how much to eat and to exercise, and how important is body shape (aesthetics) to maintaining a healthy lifestyle. No healthy person chooses to go hungry or be malnourished, but there is an *element of choice* in becoming obese. These issues are closely linked to socio-economic status, culture, and education. Eating should be enjoyable and potentially controllable, but there are often mitigating factors such as the dependability and affordability of the food supply, peer group and advertising pressures. The price of fast food sometimes makes it irresistible. In the U.S., food security among the disadvantaged is cyclical and highest around the time people get their SNAP (Supplemental Nutrition Assistance Program) food stamp dollars. Intake decreases or switches to higher calorie-per-dollar alternatives as the month progresses, when SNAP purchases run out (9). Such feast/famine cycles of food assistance may paradoxically contribute to unhealthy eating patterns.

## Parental Responsibility for Childhood Obesity?

Parents naturally want to provide for the best growth and development of their children. Parental legal rights are generally overturned to protect a minor if harm is potentially permanent and avoidable (such as refusal of life-sustaining treatment on religious grounds) (10). Obviously, obesity is serious and can lead to lifelong morbidity. The gap between chronological and biological age widens as obesity increases. Car seats, seat belts, motorcycle helmets, vaccinations, and education are nearly all universally mandated and followed, especially when freely accessible; high-quality food is not. Upstream causes, such as inappropriate overfeeding, food to placate crying, high-calorie, low-nutrient so-called addictive foods, and lack of opportunities to exercise safely in school or in home neighborhoods, all challenge the avoidability criterion.

Holding parents answerable for their child's weight implies that they have the parenting skills (which, universally are not taught) to ensure optimal nutrition quality and quantity and lifestyle for their children. Infant nutrition begins at minus 9 months and breast-feeding protects against obesity. Healthy weight children perform 13% better at school (1). Throughout childhood, parents are usually well-intentioned and serve, by example, what they eat themselves. Parents have control until the age of about 10, for their children's nutritional choices, exercise and screen time but, by adolescence, the major influencers are peer groups and social media.

## Discussion

### Integrated Action Strategies: Not More of the Same

In the final analysis, any change in body weight must follow the first law of thermodynamics. The fact that body fat mass is defended emphasizes, according to the fat cell hypothesis, the importance of early feeding practices, and parental responsibility. Obesity is caused by multifactorial bio-psycho-socio-behavioral influences; it may be inherited but it is not necessarily inevitable. Sometimes, the problem seems genetic because children adopt the eating habits and activity lifestyles of their parents. During evolution Homo sapiens has been programmed to store fat (11) and to be metabolically efficient. Metabolic efficiency actually increases as a result of weight loss and this is one of the main reasons why weight regain occurs after stopping a diet. Most of our biology as a species has evolved to survive periods of famines (with occasional feasts); but now it is ill equipped to resist the deleterious impact of a sustained surplus of food. Genes have not changed over the past 60 years (*pace* epigenetic influences); so the toxic, obesogenic environment is the main culprit for the obesity pandemic. Genes cannot be manipulated from a realistic public health or ethical standpoint. Even if we could attack the biology, say with a drug, this also is not a viable solution. For how long should it be taken—for life? What would be the economic costs? Would the people most in need of it adhere to such treatment? What about the side-effects? Medical management (including bariatric surgery) only helps re-inforce externalizing the “locus of control” without which there can be no long-term chronic disease self-management.

Human history has shown through the sad examples of wars, pestilence and economic deprivations and disparities that obesity cannot occur if food is unavailable. Therefore, we have to attack predominantly the input side of the energy equation through interventions involving the nine levels of responsibility as shown in the Table. The action points listed are not exhaustive, and must be context and time-specific, multi-level and coordinated. They do show, however, the very many options available in tailoring prevention and intervention programs to a particular setting. Preventing obesity is primarily a public health behavior and literacy issue, empowering parental and personal choices. Only when there are health complications should the medical paradigm be appropriate. There are many factors involved, including: cultural issues (norms, values, attitudes) regarding diet (especially the Mediterranean diet) and body shape; lack of knowledge about breast feeding and infant nutrition; lack of physical and economic access to quality food, especially fruits and vegetables (“food deserts,” where neighborhoods lack nearby grocery stores); lack of kitchen facilities (or time) to cook; lack of potable water which leads to over-consumption of sweetened drinks; and lack of protected, well-lit exercise facilities.

To change the environment from obesogenic to leptogenic requires government and municipal policies targeting schools, workplaces, hospitals, and public places. Interventions should be age-appropriate and involve the social media with role models, influencers, sports people, pop stars, and advertising campaigns with the same types of compelling marketing strategies that are used to sell unhealthy, calorie-rich foods. Here, health and politically correct messages are often at odds. Slogans from body-positive activists such as “fat is beautiful” or “proud to be fat” should be replaced by “fat is unhealthy and dangerous.” Smokers do not glorify their habits, neither should the obese; but, no one should be body shamed. Should cultural and identity inappropriateness prevent a thin actor/singer from adding artificial girth to play Falstaff?–of course not. We need to keep a sense of humor and proportion. However, these pressures have become more complicated. When an advertisement or social media post of a well-toned body is portrayed, the ad agency or social media poster is vilified for promoting “unrealistic expectations.” Results from the recent ACTION study (12), reporting on over 3,000 people with obesity, showed that 82% considered that weight loss was completely their own responsibility while only 5% did not agree. In this paper, “stigma” only appeared once and only in the introduction. However, stigmatization is a worldwide phenomenon with cultural differences (13). These also apply to underdiagnosed causes of obesity such as binge eating disorders (14). Addressing and avoiding stigmatization, especially in the media and social networks, are major challenges in managing and dealing with patients with obesity.

Health professionals and society must not be judgmental in treating obesity as an individual moral failing or lack of self-discipline and will power. Instead, we have to recognize that patients with obesity are also products of a society of inequality, yet we must not let society “normalize” obesity and also, at the other extreme, “too thin” models. Mis-placed medical and political correctness that leads to hands-off management of obesity, means abrogation of the physician's responsibility: it should not stop recognizing the health problems and consequences and pressing for treatment. For example, some doctors are now even reluctant to raise the issue of obesity lest they be accused of fat shaming by not accepting their patients' proportions (despite the quote at the head of this opinion piece), and thereby receive poor approval ratings in an atmosphere where popularity is equated with good healthcare.

How much involvement should there be of public health authorities, school personnel or physicians? Should there be mandatory reporting of obesity and eating disorders as for child neglect/abuse or truancy? Much depends on how measurements are made and on what follow-up programs are in place. Lifestyle education and practice should continue throughout schooling, and lunchtimes may serve as educative experiences in manners and food habits, as practiced in Norway, Japan, and elsewhere.

Legislative interventions such as sin taxes and banning soft drink vending machines and junk food advertising to children are all relevant (15). Regressive taxation may be used to benefit the population for whom it is most oppressive. Such tax revenues may go to providing parks, playgrounds, and education programs for disadvantaged children, all of which improve health outcomes. The food industry, which is part of the problem [high-calorie, nutrient-poor, hyper-palatable products (16)], must also be part of the solution by encouraging reformulations with healthier ingredients, comprehensible front-of-package food labeling and making price reductions for wholesome foods.

Suitable community-valid interventions can be based on Positive Deviant behaviors of the non-obese living in similar disadvantaged situations (17). There are also Positive Deviant countries such as Japan, Italy, and Switzerland where obesity rates are below 20%.

Obesity is one of the most difficult conditions to manage in healthcare. No-one has found the correct solution because there is no one solution. Comprehensive programs dealing with obesity require coordinated actions at all the nine levels of involvement—national, food system, educational, medical, public health, municipal, societal, parental, and individual. Parental and individual responsibility, choice and self-management clearly have a place near the center of the stage in the obesity tragedy. Otherwise, it is like going to see the play Hamlet and the Prince fails to make an appearance. Individuals are indeed responsible for their health-promoting behaviors but should be held accountable only when they have adequate resources to do so (18). In conclusion, *no one* is to be blamed, but *everyone* has a collective responsibility for working to combat the obesity pandemic—business as usual is no longer an option.

## Author Contributions

The author confirms being the sole contributor of this work and has approved it for publication.

### Conflict of Interest

The author declares that the research was conducted in the absence of any commercial or financial relationships that could be construed as a potential conflict of interest.
